# Gynaecologists’ and general surgeons’ preference for the features of integrated theatres: a discrete choice experiment

**DOI:** 10.1186/s12905-018-0576-2

**Published:** 2018-06-25

**Authors:** Tom K. Holland, Stephen Morris, Alfred Cutner

**Affiliations:** 1 Department of Women’s Health, University College Hospital, 250 Euston Road, London, NW1 2PB UK; 2 Department of Applied Health Research, University College London, Gower Street, London, WC1E 6BT UK; 3 Department of Women’s Health, University College Hospital, 250 Euston Road, London, NW1 2PB UK

## Abstract

**Background:**

Laparoscopic surgery is progressing rapidly is becoming the normal route for many abdominal operations, even for major complex surgery. The integrated laparoscopic theatre is a state-of-the-art system in which the laparoscopic equipment and multiple flat-screen monitors are permanently installed to be operational on demand inside the theatre. These expensive systems are being widely adopted, however very little research has been published regarding which features of these systems are desired by the surgeons who use them.

The study objective was to assess the strength of preference for key attributes of integrated laparoscopic theatres and to compare these preferences between Gynaecologists and General surgeons.

**Methods:**

This was an electronically distributed discrete choice experiment survey of British practicing Laparoscopic Gynaecologists and General Surgeons (Through The British Society of Gynaecology Endoscopy and The Association of Laparoscopic Surgeons of Great Britain and Ireland).

An electronic survey was designed and pre-tested. This was then sent to practicing British Laparoscopic Gynaecologists and General-Surgeons. There were structured questions regarding the seven key attributes of integrated laparoscopic theatres in the standard form for a discrete choice experiment.

**Results:**

Questionnaires from 167 respondents were analysed. One hundred three were gynaecologists and 64 were general-surgeons. Adjustable screens for height and position was the most favoured attribute and it is 4.7 times more desirable than the next most desirable attribute, which was a wire free floor. The least desirable features were piped CO2, ceiling-mounted-screens and external-transmission-of-images.

**Conclusion:**

Both groups favour adjustable screens for position and height above all the other features. These findings are in contrast with previous research, which showed that when asked to rank the attributes in order, gynaecologists chose ceiling mounted screens first and adjustable screens fourth. When asked to “trade off” attributes in the discrete choice experiment the adjustability of the screens became much more important than how the screens were mounted. With new wireless technology the benefits of a fully integrated theatre could be delivered with floor mounted systems at a considerably reduced cost. This information is important to manufacturers and purchasers of these systems in order to design cost effective ergonomic theatres that are fit for purpose.

**Electronic supplementary material:**

The online version of this article (10.1186/s12905-018-0576-2) contains supplementary material, which is available to authorized users.

## Background

Laparoscopic surgery has advanced due to improvements in both surgical technique and technical innovations. This has placed additional demands on the surgical environment and theatre design has mirrored these changing demands [1]. An ergonomic theatre design may improve team performance [2] as surgeons have been shown to have improved laparoscopic task performance when screens are directly ahead and below eye level, rather than to the side [3]. This ergonomic improvement in operating theatres reduces the risk of harm to the theatre team and patient, from potential electrical, mechanical and biological hazards [2, 4–8]. The reduction in theatre nurses’ tasks may reduce physical and mental workload and improve patient safety and job satisfaction [9]. A stressful theatre environment impairs dexterity and increases the incidence of errors [10]. Integrated theatres potentially result in a reduction in stress for the operating team [11] and enhanced efficiency during operations [12]. Thus it is likely that this less stressful theatre environment may improve patient safety. It has also been suggested that integrated theatres improve efficiency [13, 14]. However integrated theatre systems are expensive to buy and install as they require structural works to support the pendants and install the linkage between the different elements. Further hidden costs are surgical downtime during the installation process when the theatre is out of action. Average estimated costs are £0.5 million for the purchase and installation of the average high-level system but the costs can vary greatly due to different building structures that are in place before the installation. There are now alternatives to fully integrated laparoscopic theatres with floor mounted mobile systems where up to 8 screens can be wirelessly linked and positioned around the operating room as required. In addition modern camera heads enable many of the controls of a touch screen to be carried out directly from the camera head buttons. This creates a realistic alternative to the fully integrated system and may mean that the benefits of these systems can be delivered more cheaply. For these reasons it is important to understand which attributes of the currently sold laparoscopic integrated theatres are favoured by the surgeons who work in them.

A previous study [15] showed that the attributes of laparoscopic theatres which gynaecologists rated most highly, however, this study did not investigate the strength of the preference for these features, which is useful to judge how highly they are valued. In this study we chose to use a discrete choice experiment (DCE) to determine the relative importance placed on specific attributes of laparoscopic theatres as this is the best measure of how highly attributes are valued. By asking participants to choose between a series of hypothetical options with varying attributes, DCE’s reveal which attributes influence choice behaviour, establish an individual’s willingness to trade-off one attribute against another and as a result provide an insight into real-life decision making [16, 17]. The aim of this study was to assess the strength of preference for key attributes of integrated laparoscopic theatres and to compare these preferences between Gynaecologists and General surgeons.

## Methods

The study design and analysis followed current guidelines for conducting DCEs [18]. A pilot questionaire was conducted with five laparoscopic gynaecological surgeons from within the department and the findings were used to improve the finalised version of the questionnaire.

### Study sample

Participants were recruited from two specialist societies: The British Society of Gynaecology Endoscopy and The Association of Laparoscopic Surgeons of Great Britain and Ireland. Email invitations were sent out to all of the members of both societies with a link to the questionnaire on a surveymonkey™ (San Mateo, California, USA) website. One reminder email was sent to each member. Responses were anonymised but respondents were only allowed to respond once from each computer.

### Ethical approval

According to the UK National Health Service Health Research Authority online tool, ethical approval for this study was deemed unnecessary since this study was a health professional survey, which did not include invasive procedures, use of medical devices or products, human tissues or patient involvement (http://www.hra-decisiontools.org.uk/ethics).

### Questionnaire design

The questionnaire comprised 2 sections;

1. Demographic questions
2. Structured questions about the attributes of laparoscopic theatres (see Additional file 1).

The attributes used in the DCE were as follows:

1. Ceiling vs floor mounted screens
2. Screens adjustable for height or position vs fixed screens
3. Piped CO2 vs canister CO2
4. Gas on/off controlled by surgeon (sterile personnel) vs runner (non sterile personnel)
5. Room lights controlled by surgeon (sterile personnel) vs runner (non sterile personnel)
6. Wire free floors vs wires on the floors
7. External transmission of video vs none.

These were selected after previous survey-based research into this area by the authors [15] to identify the attributes of laparoscopic theatres that gynaecologists rated most highly. The attributes of laparoscopic theatres which gynaecologists rated most highly, in order, were: Ceiling mounted stacks and monitors; More than two screens; Surgeon controlling image capture/ video recording; Screens adjustable for height position and angle but may be floor mounted; Surgeon controlling laparoscope light; Touch screen control of devices by surgeon; Surgeon controlling gas flow; Surgeon controlling operating table; Piped (continuous gas flow); Surgeon controls overhead lights; Ability to transmit images to another location.

The DCE design followed the approach of Street and Burgess [19, 20]. All attributes had two levels – whether that feature was present or not. The number of possible combinations of attributes and levels were statistically reduced from 2^7^ = 128 to 8 scenarios using an orthogonal fractional main effects design [21] to give a practical number of choices for participants to complete in the questionnaire. A shift of one level was applied to the initial eight scenarios to create eight additional scenarios that were randomly paired to form eight choice sets (see Additional file 2 and Methods online). In the questionnaire participants were asked to choose one of the two options, A or B; a ‘neither’ option was not offered.

### Statistical analysis

The DCE preference data were analysed using a conditional logit regression model [22]. We anticipated positive (+) coefficients for all variables, as we expected participants to prefer each feature compared with not having it. Effects coding was used for all variables. To determine the trade-offs participants were willing to make between test attributes the marginal rates of substitution (MRS) were calculated as a ratio of the coefficients of two selected attributes. The MRS allows direct assessment of how much of one attribute participants are willing to trade for one unit of another attribute and enables a comparison of different attributes on a common scale [23]. The data were analysed for the whole group and for gynaecologists and general surgeons separately. The preferences of gynaecologists and general surgeons were compared using Wald tests.

All statistical analysis was carried out using Stata version 12.0.2 (StataCorp College Station, TX, USA).

## Results

Questionnaires from 167 respondents were analysed. One hundred three were gynaecologists (out of 639 invited 16.1%) and 64 were general surgeons (out of 533 invited 12.0%). For gynaecologists 66/103 (64.1%) were male and 37/103 (35.9%) were female and for the general surgeons these figures were 57/64 (89.1%) and 7/64 (10.9%) respectively. For both groups together this means that 123/167 (73.7%) were male and 44/167 (26.3%) were female.

For gynaecologists 72/103 (69.9%) were Consultants and 31/103 (30.1%) were Doctors in training and for the general surgeons these figures were 51/64 (79.7%) and 13/64 (20.3%) respectively. For both groups together 123/167 (73.7%) were Consultants with 44/167 (26.3%) Doctors in training.

Respondents were asked how often they carried out laparoscopic surgery. 81/167 (48.5%) performed major laparoscopic surgery every week, 31/167 (18.6%) every 2 weeks and 55/167 (32.9) once a month or less.

Table 1 shows the results for the conditional logit analysis regression for both Gynaecologists and General Surgeons combined. Adjustable screens for height and position was the most favoured attribute and it is nearly 5 times more desirable than the next most desirable attribute, which was a wire free floor (MRS = 1.584/0.335 = 4.7). The least desirable features were piped CO2, ceiling mounted screens and external transmission of images the presence or absence of which did not significantly affect participants decision-making.

**Table 1:** Conditional logit analysis regression results for Whole group *n* = 167 and Gynaecologists and General surgeons analysed separately and compared. Overall *P* = 0.02

	Whole group *n* = 167		Gynaecologists *n* = 103		General surgeons *n* = 64		Difference^a^
Attribute	Coefficient (95% CI)	*P* value	Coefficient (95% CI)	*P* value	Coefficient (95% CI)	*P* value	*P* value
Screens adjustable for height and position	1.584 (1.397 to 1.772)	< 0.0001	1.397 (1.179 to 1.615)	< 0.0001	2.046 (1.638 to 2.453)	< 0.0001	0.009
Wire free floor	0.335 (0.170 to 0.500)	< 0.0001	0.277 (0.081 to 0.474)	0.006	0.489 (0.172 to 0.807)	0.003	NS
C02 on/off controlled by sterile personnel	0.307 (0.139 to 0.474)	< 0.0001	0.247 (0.048 to 0.446)	0.015	0.477 (0.140 to 0.815)	0.006	NS
Room lights controlled by sterile personnel	0.209 (0.014 to 0.405)	< 0.0001	0.176 (−0.050 to 0.402)	0.128	0.381 (−0.044 to 0.805)	0.079	NS
Piped CO2	0.095 (−0.072 to 0.262)	NS	−0.012(− 0.211 to 0.187)	0.906	0.352 (− 0.015 to 0.689)	0.04	NS
Screens ceiling mounted	0.082 (−0.111 to 0.275)	NS	0.087 (−0.135 to 0.308)	0.444	0.092 (−0.338 to 0.522)	0.675	NS
External transmission of images	−0.006 (− 0.206 to 0.194)	NS	0.021 (− 0.210 to 0.252)	0.861	−0.033 (− 0.473 to 0.406)	0.882	NS

^a^Difference between Gynaecologist and general surgeons

Table 1 also shows the results for the conditional logit analysis regression for both Gynaecologists and General Surgeons analysed separately and compared. This shows that the most desirable four features are the same and in the same order for both groups. General surgeons were statistically significantly more likely than gynaecologists to value adjustable screens over the other attributes. The MRS is also similar for both groups (gynaecologists = 5.0, general surgeons = 4.2). Although there appears to be disagreement between the groups on the order of the least desirable 3 features, none of these significantly affected decision-making.

Table 2 shows the results for the conditional logit analysis regression for male and female participants analysed separately and compared. This shows that male and female participants agreed on the three most desirable features and in the same order. There were no significant differences when comparing the choices of male and female participants.

**Table 2:** Conditional logit analysis regression results for Male and Female participants analysed separately and compared

	Male participants *n* = 123		Female participants *n* = 44		Difference
Attribute	Coefficient (95% CI)	*P* value	Coefficient (95% CI)	*P* value	*P* value
Screens adjustable for height and position	1.61 (1.39 to 1.828)	< 0.0001	2.046 (1.638 to 2.453)	< 0.0001	NS
Wire free floor	0.321 (0.124 to 0.517)	0.001	0.367 (0.053 to 0.682)	0.022	NS
C02 on/off controlled by sterile personnel	0.314 (0.114 to 0.515)	0.002	0.290 (− 0.023 to 0.603)	0.07	NS
Room lights controlled by sterile personnel	0.176 (− 0.048 to 0.401)	0.124	0.291 (− 0.031 to 0.614)	0.076	NS
Piped CO2	0.142 (− 0.057 to 0.341)	0.162	−0.022 (− 0.336 to 0.290)	0.886	NS
Screens ceiling mounted	0.086 (− 0.141 to 0.314)	0.457	0.0676 (− 0.271 to 0.406)	0.696	NS
External transmission of images	−0.064 (− 0.302 to 0.174)	0.600	−0.138 (− 0.221 to 0.497)	0.452	NS

There were no statistically significant differences when consultants were compared with Doctors in training and when more experienced participants were compared with less experienced participants.

Table 3 shows the geographical spread of BSGE responders and BSGE members overall. Chi squared for an 8 × 2 table of difference between the location of the survey responders and the location of all BSGE members gave Chi-square = 8.93, probability *P* = 0.257. This shows that the geographical variation of the respondents was representative of the BSGE membership as a whole. Table 4 shows an example question in the DCE.

**Table 3:** Geographical spread of BSGE responders and BSGE members overall

Area	BSGE responders	BSGE overall
Wales	3	35
London	26	140
South West	13	62
South East England	14	154
Midlands	14	62
North of England	22	145
Scotland	3	30
Northern Ireland/ Ireland	3	11
Total	98	639

3 respondents did not answer this question as it was not a mandatory field and 2 responders were from outside the UK

**Table 4:** Example of a question in the DCE

In order to help improve the quality of the theatre environment for laparoscopic surgeons it is important to understand which features of laparoscopic theatres are valued and which are not valued. In the next set of questions you will be asked to choose between two different theatre configurations. Although the options may seem contrived please try to choose between the options in an honest way as this will help to evaluate the options.	
Please choose option A or B.	
Option A.	
The screens and stacks are floor mounted. The screens are not adjustable for height, angle or position. The C02 is in canisters. The laparoscopy light, CO2 gas on/ off and room lights are controlled by the non scrubbed nursing staff. There are wires on the floor and there is no external transmission of the images.	
Option B.	
The stacks and screens are ceiling mounted and fully adjustable for height, angle and position. The CO2 is piped (continuous) and the sterile personnel (surgeon or scrub nurse) can control the laparoscope light, CO2 gas on/off and the overhead room lights. The floor is wire free and the there is external transmission of video set up.	

## Discussion

This is the first study to use a discrete choice experiment to assess the features of integrated laparoscopic theatres that surgical personnel favour. It is also the first study to compare different choices that exist between general surgeons and gynaecologists regards laparoscopic theatres.

The integrated laparoscopic theatre is a state-of-the-art system in which multiple flat-screen monitors and other laparoscopic equipment are permanently installed to be operational on demand inside the theatre. This system facilitates versatile positioning of equipment as they are installed on columns attached to a ceiling-mounted suspension system. All the wiring is concealed inside the suspension system and led out through the ceiling which reduces operating room clutter and improves safety [4, 24]. The CO2 gas, rather than utilising small canisters, is piped on demand thus reducing the time needed to change canisters and the resultant loss of a pneumoperitoneum that goes with loss of gass. The surgeon and theatre staff are able to control the laparoscopic and theatre room equipment using touch screen monitors. This is in contrast to the traditional laparoscopic systems with one, non adjustable screen which is mounted on top of a floor mounted stack of equipment which can be wheeled into theatre as required.

Generally there was agreement between general surgeons and gynaecologists with the same four attributes favoured in the same order. Both groups favour adjustable screens for position and height above all the other features with general surgeons more strongly favouring this attribute than gynaecologists. These findings are in contrast with the findings of our previous study, which showed that when asked to rank the attributes in order, gynaecologists chose ceiling mounted screens first and adjustable screens fourth. When asked to “trade off” attributes in the discrete choice experiment the adjustability of the screens became much more important than how the screens were mounted.

The findings of our study suggest that the main benefit of integrated laparoscopic theatres is the ability to position the screens almost anywhere in the theatres. Due to wireless technology, floor mounted systems could be designed to function in the same way as current integrated theatres. This removes the need for expensive building works and long theatre down time during installation. Thus the modern laparoscopic theatre could be delivered at a considerably reduced cost. This information is important to manufacturers and purchasers of these systems in order to design cost effective ergonomic theatres that are fit for purpose. Thus the advent of wireless connectivity technology with monitors on slim versatile stands enables the market place to be expanded at a much reduced cost.

### Strengths and limitations of the study

This is the first study to use a discrete choice experiment to assess the desirability of the features of integrated laparoscopic theatres. The relatively small sample size is a limitation of the study. Another limitation of the study is that not all responders have regular access to fully integrated theatres.

Further research on this area, especially among a more international cohort and other surgical specialties, would be helpful to improve our understanding of this issue.

## Conclusion

Both groups favour adjustable screens for position and height above all the other features. These findings are in contrast with previous research, which showed that when asked to rank the attributes in order, gynaecologists chose ceiling mounted screens first and adjustable screens fourth. When asked to “trade off” attributes in the discrete choice experiment the adjustability of the screens became much more important than how the screens were mounted. With new wireless technology the benefits of a fully integrated theatre could be delivered with floor mounted systems at a considerably reduced cost. This information is important to manufacturers and purchasers of these systems in order to design cost effective ergonomic theatres that are fit for purpose.

## Additional files

Additional file 1: Full data set as a PDF. (PDF 120 kb)

Additional file 2: Full data set as a excel spreadsheet. (XLS 69 kb)

## Abbreviations

CO2: Carbon dioxide
DCE: Discrete choice experiment
MRS: Marginal rates of substitution
