# Supplementary material for: Gynaecologists’ and general surgeons’ preference for the features of integrated theatres: a discrete choice experiment
Source: BMC Womens Health. 2018 Jun 25;18:112. doi: 10.1186/s12905-018-0576-2 (PMC6019828; doi:10.1186/s12905-018-0576-2)
Supplement: Supplementary file 1 — Full data set as a PDF. (PDF 120 kb) [file 12905_2018_576_MOESM1_ESM.pdf]

1. Are you male or female?

- ◯ Male
- ◯ Female

* 2. What grade are you?

- ◯ Consultant
- ◯ Associate specialist
- ◯ Specialist trainee (SHO) year 1-2
- ◯ Specialist trainee (Junior registrar) year 3-5
- ◯ Specialist trainee (Senior registrar) year 6+
- ◯ other

Other (please specify)

3. Which region do you work in?

* 4. How many years have you been operating laparoscopically?

- ◯ 0-5
- ◯ 5-10
- ◯ 10-15
- ◯ 15-20
- ◯ 20+

* 5. How often do you operate laparoscopically?

- ◯ every week
- ◯ every 2 weeks
- ◯ every month
- ◯ every 2 months
- ◯ rarely

* 6. How often do you perform major laparoscopic surgery such as TLH, Laparoscopic myomectomy etc?

- ◯ every week
- ◯ every 2 weeks
- ◯ every month
- ◯ every 2 months
- ◯ rarely

* 7. How many hours on average do you spend performing laparoscopic surgery each week?

- ◯ 1-2
- ◯ 3-5
- ◯ 6-10
- ◯ 11-15
- ◯ 15+

* 8. Have you ever operated in an integrated theatre (with for example ceiling mounted screens and stacks, surgeon controlled gas, lights, etc)

- ◯ Never
- ◯ Occasionally (1-6 times per year)
- ◯ Regularly (>6 times per year)

* 9. If you have used an integrated theatre was this in:

- ◯ an NHS hospital
- ◯ a private hospital
- ◯ at an industry product demonstration

* 10. In order to help improve the quality of the theatre environment for laparoscopic surgeons it is important to understand which features of laparoscopic theatres are valued and which are not valued.

In the next set of questions you will be asked to choose between two different theatre configurations. Although the options may seem contrived please try to choose between the options in an honest way as this will help to evaluate the options.

Please choose option A or B.

Option A.
The screens and stacks are floor mounted.
The screens are not adjustable for height, angle or position.
The C02 is in canisters.
The laparoscopy light, CO2 gas on/ off and room lights are controlled by the non scrubbed nursing staff.
There are wires on the floor and there is no external transmission of the images.

Option B.
The stacks and screens are ceiling mounted and fully adjustable for height, angle and position.
The CO2 is piped (continuous) and the sterile personnel (surgeon or scrub nurse) can control the laparoscope light, CO2 gas on/off and the overhead room lights.
The floor is wire free and the there is external transmission of video set up.

- ○ Option A.
- ○ Option B.

* 11. Please choose option A or B

Option A.

The stacks and screens are floor mounted and are not adjustable for height, angle or position.
The C02 is in canisters and the sterile personnel (surgeon or scrub nurse) can control the laparoscope light, CO2 gas on/off and the overhead room lights.
The floor is wire free and there is external transmission of video set up.

Option B.

The stacks and screens are ceiling mounted and fully adjustable for height, angle and position.
The CO2 is piped (continuous).
The laparoscopy light, CO2 gas on/ off and room lights are controlled by the non scrubbed nursing staff.
There are wires on the floor and there is no external transmission of the images.

- ○ Option A.
- ○ Option B.

* 12. Please choose option A or B

Option A.

The stacks and screens are floor mounted and are fully adjustable for height, angle and position.
The CO2 is piped (continuous).
The laparoscopy light, CO2 gas on/ off and room lights are controlled by the non scrubbed nursing staff.
The floor is wire free and there is external transmission of video set up.

Option B.

The stacks and screens are ceiling mounted.
The screens are not adjustable for height, angle or position.
The C02 is in canisters.
The laparoscopy light, CO2 gas on/ off and room lights are controlled by the non scrubbed nursing staff.
The floor is wire free and there is external transmission of video set up

- ◯ Option A
- ◯ Option B

* 13. Please choose option A or B.

Option A.
The stacks and screens are floor mounted.
The screens are fully adjustable for height, angle and position.
The CO2 is piped (continuous).
The sterile personnel (surgeon or scrub nurse) can control the laparoscope light, CO2 gas on/off and the overhead room lights.
There are wires on the floor and there is no external transmission of the images.

Option B
The stacks and screens are ceiling mounted.
The screens are not adjustable for height, angle or position.
The C02 is in canisters.
The laparoscopy light, CO2 gas on/ off and room lights are controlled by the non scrubbed nursing staff.
There are wires on the floor and there is no external transmission of the images.

- ◯ Option A.
- ◯ Option B.

* 14. Please choose option A or B

Option A.
The stacks and screens are ceiling mounted.
The screens are not adjustable for height, angle or position.
The CO_2 is piped (continuous).
The room lights are controlled by the non scrubbed nursing staff.
The sterile personnel (surgeon or scrub nurse) can control the laparoscope light and CO_2 gas on/off. There are wires on the floor and there is external transmission of video set up.

Option B.
The stacks and screens are floor mounted.
The screens are fully adjustable for height, angle and position.
The C02 is in canisters.
The laparoscope light and CO_2 gas on/off are controlled by the non scrubbed nursing staff.
The sterile personnel (surgeon or scrub nurse) can control the room lights.
The floor is wire free and there is no external transmission of video.

- ◯ Option A
- ◯ Option B

* 15. Please choose option A or B.

Option A.

The stacks and screens are ceiling mounted.
The screens are not adjustable for height, angle or position.
The CO_2 is piped (continuous).
The sterile personnel (surgeon or scrub nurse) can control overhead room lights.
The laparoscope light and CO_2 gas on/off are controlled by the non scrubbed nursing staff.
The floor is wire free and there is no external transmission of video.

Option B.
The stacks and screens are floor mounted.
The screens are fully adjustable for height, angle and position.
The C02 is in canisters.
The overhead room lights are controlled by the non scrubbed nursing staff.
The sterile personnel (surgeon or scrub nurse) can control the laparoscopy light, CO_2 gas on/ off.
There are wires on the floor and there is external transmission of video.

- ◯ Option A
- ◯ Option B

* 16. Please choose option A or B.

Option A.
The stacks and screens are ceiling mounted.
The screens are fully adjustable for height, angle and position.
The C02 is in canisters.
The overhead room lights are controlled by the non scrubbed nursing staff.
The sterile personnel (surgeon or scrub nurse) can control the laparoscopy light, CO2 gas on/ off.
The floor is wire free and there is no external transmission of video.

Option B.
Floor mounted stacks and screens.
The screens are not adjustable for height, angle or position.
The CO2 is piped (continuous).
The sterile personnel (surgeon or scrub nurse) can control overhead room lights.
The laparoscope light and CO2 gas on/off are controlled by the non scrubbed nursing staff.
There are wires on the floor and there is external transmission of video.

- ◯ Option A.
- ◯ Option B.

* 17. Please choose option A or B

Option A.
The stacks and screens are ceiling mounted.
The screens are fully adjustable for height, angle and position.
The C02 is in canisters.
The laparoscope light and CO2 gas on/off are controlled by the non scrubbed nursing staff.
The sterile personnel (surgeon or scrub nurse) can control the room lights.
There are wires on the floor and there is external transmission of video set up.

Option B.
The stacks and screens are floor mounted.
The screens are not adjustable for height, angle or position.
The CO2 is piped (continuous).
The room lights are controlled by the non scrubbed nursing staff.
The sterile personnel (surgeon or scrub nurse) can control the laparoscope light and CO2 gas on/off.
The floor is wire free and there is no external transmission of video.

- ◯ Option A
- ◯ Option B
